# Heat shock protein 60 (HSP60) modulates adiponectin signaling by stabilizing adiponectin receptor

**DOI:** 10.1186/s12964-020-00546-5

**Published:** 2020-04-09

**Authors:** Deling Zhang, Hua Liu, Yemin Zhang, Junfeng Li, Yalin Fu, Yuyang Zheng, Jie Wu, Mingke Ma, Zhongyuan Wen, Changhua Wang

**Affiliations:** 1 Department of Pathology & Pathophysiology, Wuhan University School of Basic Medical Sciences, Wuhan, 430071 China; 2 Hubei Provincial Key Laboratory of Developmentally Originated Disease, Wuhan, 430071 China; 3 Department of Clinical Pathology, The First People’s Hospital of Lianyungang, Lianyungang, 222061 China; 4 Department of Endocrinology, Renmin Hospital of Wuhan University, Wuhan, 430060 China

**Keywords:** Heat shock protein 60, Adiponectin, Adiponectin receptor, Cardiac myocyte

## Abstract

Adiponectin, an adipokine produced and secreted by adipocytes, is involved in regulating the development and progression of insulin resistance, diabetes, and diabetic complications. Heat shock protein 60 (HSP60) is a molecular chaperone, most commonly presenting in mitochondria and participating in the maintenance of protein homeostasis. Accumulating studies have demonstrated that the elevated circulating HSP60 and the decreased intracellular HSP60 are closely associated with diabetic complications such as diabetic cardiomyopathy. However, the underlying mechanism remains poorly understood. In the present study, we reported that HSP60 interacted directly with adiponectin receptors. Its abundance was positively associated with adiponectin action. Furthermore, HSP60 depletion markedly mitigated the protective impacts of adiponectin on high glucose-induced oxidative stress and cell apoptosis in rat cardiac H9c2 cells. In addition, HSP60 knockdown significantly enhanced proteasome activity leading to the degradation of adiponectin receptor 1. Taken together, we showed for the first time that HSP60 interacted with adiponectin receptors and mediated adiponectin signaling through stabilizing adiponectin receptor. This in vitro study also provides an alternative explanation for mechanism by which adiponectin exerts its action.

**Video abstract**

**Video abstract**

## Background

Adiponectin is the most abundant adipokine produced and secreted by adipocytes. Through binding with its specific receptors adiponectin receptor 1 (AdipoR1) and AdipoR2, adiponectin initiates intracellular signaling pathways and exerts promising effects in the prevention or treatment of diabetes and metabolic syndrome, cardiovascular diseases, cancers, central nervous system disorders and so on [1–4]. Previous researches have confirmed that adiponectin signaling could be mediated by adaptor protein APPLs (adaptor protein, phosphotyrosine interacting with PH domain and leucine zipper) including APPL1 and APPL2 [5, 6]. APPL1 associates with the intracellular domain of AdipoRs and positively regulates adiponectin’s actions in some type of cells such as sensitizing insulin signaling in skeletal muscle cells [5, 6]. APPL1-deficiencies in mice impair adiponectin signaling and therefore cause systemic insulin resistance [7]. In contrast, APPL2 negatively regulates adiponectin signaling by competitively interacting with AdipoRs or heterodimerizing with APPL1 [6]. The “Yin and Yang” balance between APPL1 and APPL2 orchestrates adiponectin signaling and maintains normal adiponectin function [6, 8].

Heat shock protein 60 (HSP60) is classically described as a molecular chaperone, most commonly presenting in mitochondria and involving in the maintenance of protein homeostasis. Under stress condition, HSP60 can translocate to the cytosol and cell membrane, and also secrete into blood to form serum (or circulating) HSP60 [9]. The ability of HSP60 in response to different stress greatly dependents on its localization [10]. It is noteworthy that there is an interaction between HSP60 and inflammation. HSP60 expression and secretion can be promoted in viable cells such as cardiomyocytes, adipocytes, astrocytes, and peripheral blood mononuclear cells, in response to proinflammatory cytokines as diverse as IL-1β and TNF-α [11–14]. On the other hand, serum HSP60 has been recognized as a potent inductor of proinflammatory mediators in various cells indulging innate immune cells, skeletal muscle, cardiomyocytes, and adipocytes [11, 15–17]. Furthermore, the high levels of serum HSP60 have been found in the individuals with adjuvant arthritis and atherosclerosis [18, 19].

Currently, accumulating evidences have linked HSP60 with diabetes mellitus and diabetic complications, although the molecular mechanisms are poorly understood [18–20]. For instance, serum HSP60 levels have been found to be significantly elevated in the patients with type 2 diabetes and morbid obesity, due to enhanced mitochondrial stress and responsible for inflammation [20–22]. A modified form of highly reactive HSP60 peptide p277 (DiaPep277) has been testing to treat type 1 diabetes [23]. In addition, the elevated serum HSP60 levels also increases cardiovascular risk in obesity individuals [22]. Therefore, HSP60 may represent a potential therapeutic target for diabetes and its complications.

Interestingly, type 2 diabetic subjects exhibit the decreased expression of intracellular HSP60 in some tissues such as brain, heart, and subcutaneous adipose tissue [24–26]. Importantly, the decrease in intracellular HSP60 levels is closely associated with inflammation, mitochondrial dysfunction, formation of reactive oxygen species (ROS), and insulin resistance, which are usually observed in diabetic individuals and prevented by adiponectin administration [4, 24–27]. However, the status of HSP60 in adiponectin signaling is unclear.

Here, we experimentally demonstrated that HSP60 mediated adiponectin signaling in vitro by stabilizing adiponectin receptor. This finding will undoubtedly help us to deepen our understanding of adiponectin action and explore a novel therapy strategy for diabetes and diabetic complications.

## Materials and methods

### Antibodies and reagents

Antibodies against to AMPKα (#5831), phospho-AMPKα (Thr172) (#2535), p38 MAPK (#8690), phospho-p38 MAPK (Thr180/Tyr182) (#9216), caspase-3 (#9662), cleaved caspase-3 (#9661), Myc-tag (#2276), ubiquitin (#3936), β-tubulin (#2146) were from Cell Signaling Technology (Billerica, MA, USA). Antibodies against to AdipoR1 (ab70362), AdipoR2 (ab77612), and HSP60 (ab46798) were obtained from Abcam (Cambridge, MA, USA). Normal IgG (sc-2025) and secondary antibodies conjugated to horseradish peroxidase or alkaline phosphatase were purchased from Santa Cruz Biotechnology (Santa Cruz, CA, USA) or Abbiotec (San Diego, CA, USA), respectively. Recombinant mouse adiponectin (ALX-522-059) and recombinant rat adiponectin globular form (Catalog#: SRP4593) were acquired from Enzo Life Sciences (Farmingdale, NY, USA) and Sigma-Aldrich (St. Louis, MO, USA), respectively. MG132 (HY-13259) was obtained from MedChemExpress (Monmouth Junction, NJ, USA).

### Cell culture and treatment

Rat cardiac H9c2 cell (ATCC, CRL-1446) were cultured in DMEM containing 10% fetal bovine serum (FBS) and 1% penicillin/streptomycin. Mouse liver HepIR cells (kind gifts from Drs. Feng Liu and Lily Q. Dong, UTHSCSA, USA) were cultured in MEM-alpha containing 10% FBS and 0.8 μM dexamethasone [6, 28]. All cells were maintained in a humidified incubator with 5% CO_2_ and 95% air at 37 °C.

High glucose treatment was performed as our described previously [29, 30]. The control group received the treatment of 5.5 mM glucose and the identical concentration of mannitol which act as osmotic control to remove a hyperosmolar effect.

### Plasmid construction

The cDNAs of full-length of mouse HSP60, mouse AdipoR1, and mouse AdipoR2 were generated by PCR and subcloned into the mammalian expression vectors pcDNA3.1 (Myc-tagged), or pGEX, respectively, as described previously [6].

### Small interfering RNAs and transfection

The small interfering RNAs (siRNAs) targeting rat HSP60 (NM_022229.2) and mouse HSP60 (NM_010477.4) were synthesized by Genechem Co., LTD (Shanghai, China). Transfection was performed with 120 pM of siRNA using Lipofectamine® RNAiMAX Transfection Reagent (Life Technologies Corporation, Gaitherburg, MD, USA) according to the manufacturer’s protocol. The most effective sequences of siRNAs and its paired control used in the experiments were as follows: rat HSP60, 5′- GAGAGGTGTGATGTTGGCTGTTGAT-3′ and 5′- GAGTGTGGTAGGGTTTGTCTGAGAT − 3′; mouse HSP60, 5′-CAAATGGAGACAAAGACATTGGGAA-3′ and 5′-CAAAGGCAGAAACAGTTAGGATGAA-3′. Knockdown efficiency was assessed by western blot.

### Cell immunofluorescence

Immunofluorescence staining was performed as our described previously [6, 30]. Images were acquired on an Olympus IX83 laser scanning confocal microscope and analyzed by Olympus FV1200 software.

### DHE staining

The real-time formation of ROS in cells was detected by dihydroergotamine (DHE) staining as described previously [30]. Briefly, the cells were plated on the coverslips within a 24-well plate at a density of 2 × 10^4^ cells/well, starved serum for 6 h, and then treated with or without high glucose and/or other compound for the desired time. DHE (at a final concentration of 10 mM) was used to stain the cells at 37 °C for 30 min in the dark. Cells were then rinsed once with pre-warmed PBS. DHE fluorescence was captured with fluorescence microscopy and quantified by automated image analysis.

### Apoptosis determination

Terminal deoxynucleotidyl transferase-mediated dUTP-biotin nick end labeling (TUNEL) was performed to detect cells undergoing apoptosis as described by the manufacturer’s protocol (Roche Applied Science, Indianapolis, IN, USA).

### GST pull-down, immunoprecipitation and western blot

The pull-down assay, immunoprecipitation experiments, and western blot were performed as described previously [6].

### Statistical analyses

The data are presented as the means ± SD. Differences between the groups were examined using one-way analysis of variance (ANOVA), followed by a Newman-Keuls post hoc test. The values of *p* < 0.05 were considered statistically significant.

## Results and discussion

### HSP60 associated with adiponectin receptors

**Fig. 1:**
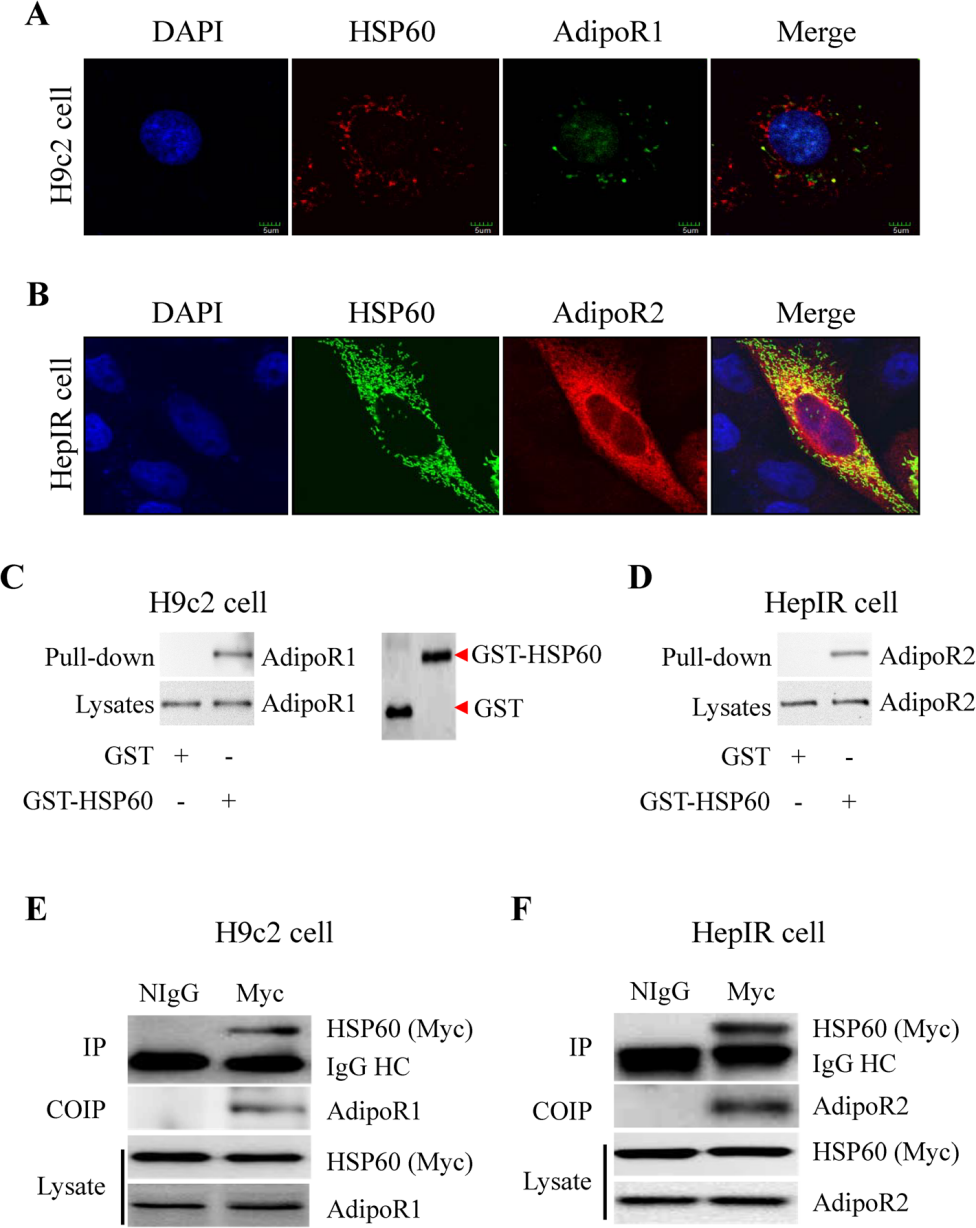
HSP60 interacted with adiponectin receptors. **a** Colocalization of HSP60 and AdipoR1 in H9c2 cells. **b** Colocalization of HSP60 and AdipoR2 in HepIR cells. **c** Left: Pull-down of endogenous AdipoR1 with GST-HSP60 in H9c2 cells; Right: Western blot analysis of GST or GST-HSP60. **d** Pull-down of endogenous AdipoR2 with GST-HSP60 in HepIR cells. **e** Coimmunoprecipitation of AdipoR1 with HSP60 in H9c2 cells. **f** Coimmunoprecipitation of AdipoR2 with HSP60 in HepIR cells

Adiponectin receptors, AdipoR1 and AdipoR2, are important members in a new family of cell surface receptor, called Progestin and AdipoQ Receptor (PAQR) family [31]. AdipoR1 is expressed ubiquitously and constitutively in most tissues and cells including adult cardiomyocytes and rat cardiac H9c2 cells [32, 33], while AdipoR2 is mainly expressed in the liver [34]. To demonstrate the association between AdipoRs with HSP60, we firstly detected the localization of AdipoRs and HSP60 in H9c2 cells and mouse liver HepIR cells [28]. Immunofluorescence staining revealed that endogenous HSP60 co-localized with endogenous AdipoR1 in H9c2 cells (Fig. 1a). Similar observation was also made when HSP60 was overexpressed in HepIR cells (Fig. 1b). To investigate whether HSP60 and AdipoRs associate directly, GST pull-down and co-immunoprecipitation assays were performed. As shown in Fig. 1c and d, endogenous AdipoR1 in H9c2 cells and AdipoR2 in HepIR cells interacted with GST-HSP60 but not with GST control proteins. Co-immunoprecipitation experiments revealed that overexpressed HSP60 interacted specifically with endogenous AdipoR1 in H9c2 cells (Fig. 1e) and endogenous AdipoR2 in HepIR cells (Fig. 1f). These findings indicate that HSP60 interacts directly with adiponectin receptors.

### HSP60 mediated adiponectin action

**Fig. 2:**
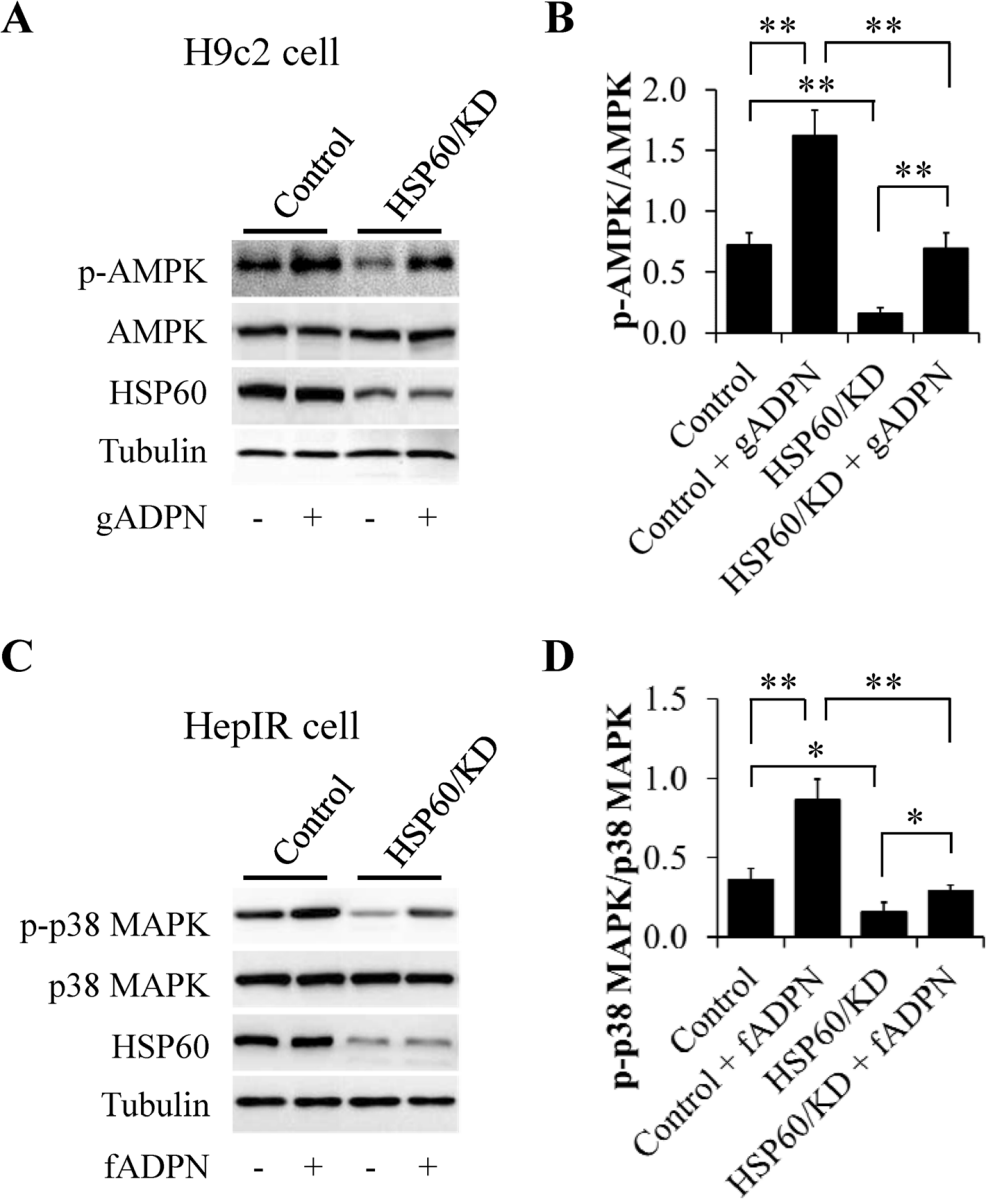
HSP60 knockdown attenuated adiponectin action. **a** Effects of HSP60 knockdown on adiponectin-stimulated phosphorylation of AMPK in H9c2 cells. **b** Quantification of phosphor-AMPK/AMPK in (**a**). **c** Effects of HSP60 knockdown on adiponectin-stimulated phosphorylation of p38 MAPK in HepIR cells. **d** Quantification of phosphor-p38 MAPK/p38 MAPK in (**c**). Results are mean ± SD. *n* = 4. *P < 0.05, **P < 0.01 compared with the indicated group (one-way ANOVA)

To understand the functional role of HSP60 in regulating adiponectin action, intracellular HSP60 protein levels were increased by overexpression (OE) or decreased by siRNA knockdown (KD), respectively. The cells were then starved serum for 6 h, followed by stimulation with 1 μg/ml adiponectin for 30 min. It has been reported that AdipoR1-mediated adiponectin signaling could be activated by globular adiponectin (gADPN) whereas AdipoR2 only bind with full-length adiponectin (fADPN) [31, 34]. Therefore, H9c2 cells and HepIR cells were treated with gADPN and fADPN, respectively. We found that phosphorylation of AMPK and p38 MAPK in response to adiponectin stimulation were greatly suppressed in HSP60-KD H9c2 cells (Fig. 2a and b) and HepIR cells (Fig. 2c and d) but obviously enhanced in HSP60-OE H9c2 cells (Additional file 1: Figure S1a and S1b) and HepIR cells (Additional file 1: Figure S1c and S1d), respectively. Since phosphorylation of AMPK in cardiomyocytes and p38 MAPK in hepatocytes are the markers of their activities [35, 36], our results demonstrate that HSP60 positively modulates adiponectin signaling.

In the present study, we also found that knocking down of HSP60 induced reductions of p38 MAPK and AMPK phosphorylation at basal levels (Fig. 2). Although the underlying mechanism is unclear, HSP60 has been proven to positively regulate p38 MAPK pathway in various cells [37, 38], suggesting that HSP60 plays a role in controlling p38 MAPK activity in both adiponectin-dependent and -independent mechanism. The impacts of HSP60 on AMPK activity is controversial. In cancer cells, HSP60 silencing can activate AMPK through triggering the excessive ROS production, which is beneficial for tumor progression [39, 40]. In adipose tissues, however, high-fat diet feeding induces a reduction of HSP60 protein levels and this change is not associated with any changes in AMPK activity [41]. Our finding indicates that HSP60 deficiency reduced basal AMPK phosphorylation, suggesting that adiponectin-independent mechanism is also involved in HSP60 controlled AMPK activation. Future studies are needed to dissect the specific role of HSP60 in variety of the cells residing in fat tissues in regulating AMPK activity.

### HSP60 knockdown mitigated the protective effects of adiponectin on high glucose-induced oxidative stress and cell apoptosis in H9c2 cells

Hyperglycemia is a hallmark feature of both type 1 and type 2 diabetes. Previous study has evidenced that high levels of glucose induce oxidative stress and cell apoptosis in cardiomyocytes [30, 42], which can be protected by adiponectin administration [43]. Using this model, we wanted to further confirm the role of HSP60 in mediating adiponectin signaling.

H9c2 cells were starved serum for 6 h, and then incubated with 5.5 mM (normal glucose control) or 33 mM glucose (high glucose, HG) in the presence or absence of 1 μg/ml of gADPN for another 48 h. TUNEL and DHE staining assays were carried out to detect cell apoptosis and real-time formation of ROS, respectively. The cleaved caspase-3 was detected by western blot to confirm the progression of apoptosis.

**Fig. 3:**
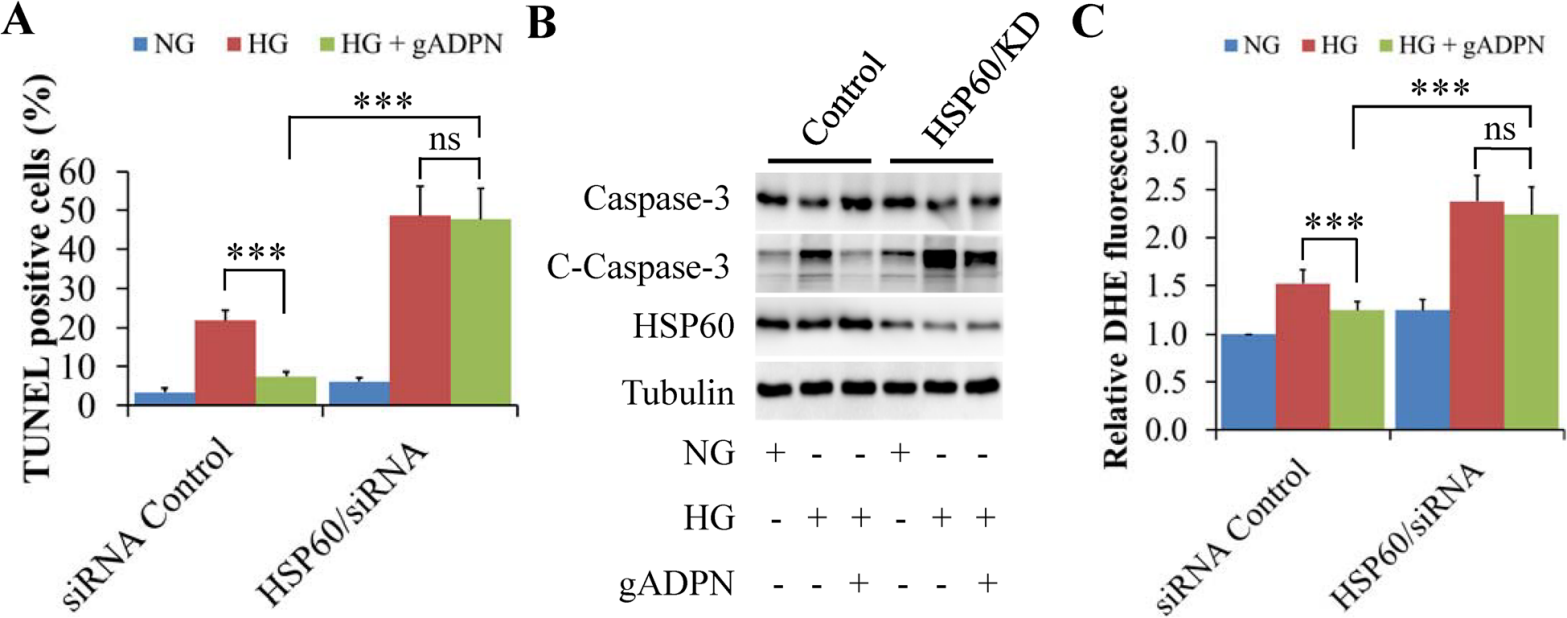
HSP60 knockdown abolished the protective effects of adiponectin on high glucose-induced apoptosis and ROS formation in cardiac H9c2 cells. **a** Quantification of cell apoptosis. **b** Effects of HSP60 knockdown on cleaved caspase-3 levels. **c** Quantification of DHE staining. Results are mean ± SD. *n* = 4. ***P < 0.001 compared with the indicated group (one-way ANOVA). ns: no statistical significance

We found that HSP60 depletion significantly increased cell apoptosis, even on normal glucose (Fig. 2a, Additional file 2: Figure S2a, and Fig. 3b). This finding is consistent with previous study showing that the deletion of HSP60 in adult cardiomyocytes results in the impairment of structure and function of cardiac muscle cells [44]. Furthermore, adiponectin administration markedly inhibited HG-induced apoptosis in siRNA control cells (Fig. 3a, Additional file 2: Figure S2a, and Fig. 3b). However, these protective effects were almost completely diminished in HSP60-KD cells (Fig. 3a, Additional file 2: Figure S2a, and Fig. 3b). The similar effects on ROS formation were found in siRNA control or HSP60-KD cells treated with or without adiponectin (Fig. 3c and Additional file 2: Figure S2b). These findings further confirm the HSP60 regulation on adiponectin signaling.

### HSP60 stabilized adiponectin receptor through a proteasome-dependent mechanism

**Fig. 4:**
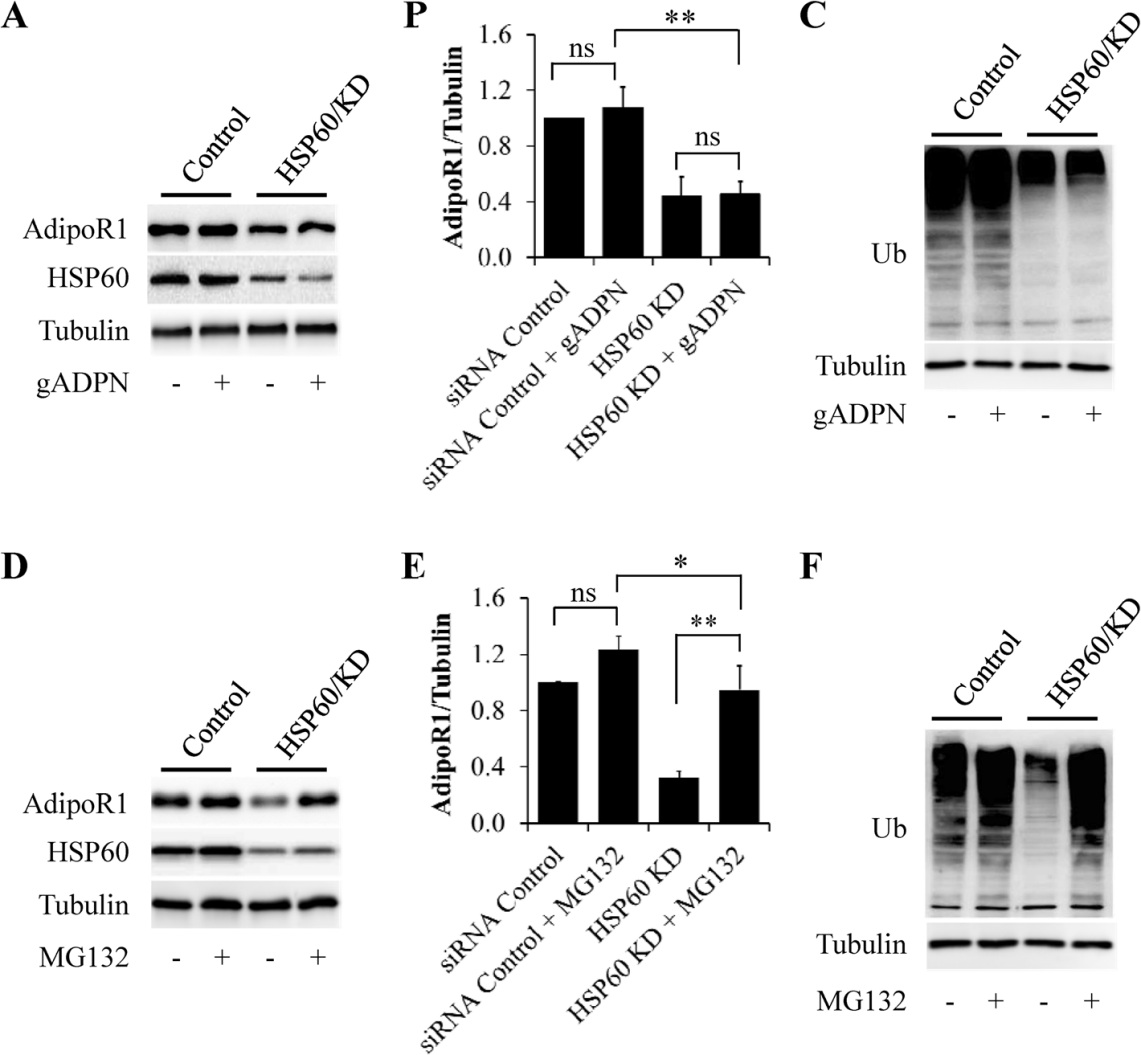
HSP60 knockdown reduced AdipoR1 levels in cardiac H9c2 cells. **a** Effects of HSP60 knockdown on AdipoR1 expression. **b** Quantification of AdipoR1 protein levels in (**a**). **c** Effects of HSP60 knockdown on the ubiquitination of total proteins. **d** Effects of proteasome inhibitor MG132 on HSP60 knockdown-induced reduction of AdipoR1 expression. **e** Quantification of AdipoR1 protein levels in (**d**). **F** Effects of MG132 on the ubiquitination of total proteins. Results are mean ± SD. n = 4. *P < 0.05, **P < 0.01 compared with the indicated group (one-way ANOVA). ns: no statistical significance

HSP60 has been found to positively regulate insulin-like growth factor-1 (IGF-1) signaling, through maintaining the abundance of IGF-1 receptor in cardiac muscle cells [45]. To figure out whether the similar mechanism exists in adiponectin receptors, we observed the effects of HSP60 KD on the protein levels of AdipoR1 in cardiac H9c2 cells. The cells were starved serum for 6 h, followed by stimulation with 1 μg/ml of gADPN for 18 h. We found that AdipoR1 expression was significantly reduced by HSP60 depletion but not affected by adiponectin treatment (Fig.4a and Fig. 4b), suggesting that HSP60 depletion induced AdipoR1 degradation.

It is well-known that intracellular protein degradation is mainly induced by two cellular routes: the ubiquitin-proteasome system (UPS) and the autophagy-lysosome system [46]. HSP60 has been reported to modulate proteasome activity and protein ubiquitination [45, 47]. We thus investigated the potential effects of HSP60 on the UPS. Indeed, HSP60 depletion markedly decreased the ubiquitination of total proteins (Fig. 4c). In addition, 20S proteasome activity was also greatly enhanced in HSP60-KD H9c2 cells (Additional file 3: Figure S3). Consistent with study performed in yeast [47], these findings demonstrate that HSP60 can inhibit proteasome activity in the mammalian cells.

When H9c2 cells were starved serum for 6 h, followed by incubation with 0.1 μM of MG132, a specific proteasome inhibitor for 18 h, we found that proteasome inhibition significantly restored HSP60 depletion-reduced protein levels of AdipoR1 (Fig. 4d and e). Proteasome inhibition also significantly increased the ubiquitination of total proteins when compared with HSP60-KD cells (Fig. 4f). These findings further suggested that HSP60 depletion-induced AdiopR1 degradation is mediated by a proteasome-dependent mechanism.

## Conclusion

Adiponectin resistance, namely reduced biologic response to adiponectin in adiponectin-sensitizing tissues or cells, such as adipocytes, skeletal muscle, liver, the vasculature, and the heart [48–50], is closely associated with the development and progression of obesity, diabetes, inflammation, atherosclerosis, and cardiovascular diseases [51]. Accumulating studies have demonstrated that adiponectin resistance is related to decreased adiponectin receptor expression, reduced receptor sensitivity, and dysfunctional downstream signaling [48–50]. However, the mechanism underlying adiponectin receptor downregulation remains elusive. In the present study, we showed for the first time that HSP60 interacted with adiponectin receptors and mediated adiponectin signaling. It is highly notable that HSP60 could stabilize AdipoR1 expression through suppressing proteasome activity. This in vitro study provided an alternative explanation for the mechanism underlying adiponectin action. Given that the alteration of HSP60 protein levels have been demonstrated in diabetic complications and functionally related to hyperglycemia-induced cell injury [19], our findings will also advance our insights into basic mechanisms of HSP60 function.

In addition, our results suggest that HSP60 might represent a promising therapeutic opportunity in the diabetic complications such as diabetic cardiomyopathy. However, more in vitro and in vivo studies are necessary to further confirm our findings and to gain a full understanding of HSP60 relevance. It also needs to evaluate the impacts of HSP60 on AdipoR2 expression and figure out its clinical significance.

## Supplementary information

**Additional file 1 Figure S1.** HSP60 overexpression enhanced adiponectin action. **A** Effects of HSP60 overexpression on adiponectin-stimulated phosphorylation of AMPK in H9c2 cells. **B** Quantification of phosphor-AMPK/AMPK in (A). **C** Effects of HSP60 overexpression on adiponectin-stimulated phosphorylation of p38 MAPK in HepIR cells. **D** Quantification of phosphor-p38 MAPK/p38 MAPK in (C). Results are mean ± SD. *n* = 4. **P < 0.01, ***P < 0.001 compared with the indicated group (one-way ANOVA).

**Additional file 2: Figure S2.** Effects of HSP60 knockdown on cell apoptosis and ROS formation in cardiac H9c2 cells. **A** Representative images showing the effects of HSP60 knockdown on apoptosis. **B** Representative images showing the effects of HSP60 knockdown on ROS formation.

**Additional file 3: Figure S3.** Effects of HSP60 knockdown on 20S proteasome activity in cardiac H9c2 cells. siRNA control and HSP60 KD cells were starved serum for 6 h. The chymotrypsin-like activity of 20S proteasome was determined using synthetic fluorogenic peptide substrate Suc-LLVY-AMC as described previously [52]. Results are mean ± SD. *n* = 4. ***P < 0.001 compared with the siRNA control group (one-way ANOVA).

## Data Availability

All supporting data are included in this published article.

## Abbreviations

HSP60: Heat shock protein 60
AdipoR1: Adiponectin receptor 1
ROS: Reactive oxygen species
TUNEL: Terminal deoxynucleotidyl transferase-mediated dUTP-biotin nick end labeling
UPS: Ubiquitin-proteasome system
HG: High glucose
