# Supplementary figures and images for: Heat shock protein 60 (HSP60) modulates adiponectin signaling by stabilizing adiponectin receptor
Source: Cell Commun Signal. 2020 Apr 9;18:60. doi: 10.1186/s12964-020-00546-5 (PMC7147001; doi:10.1186/s12964-020-00546-5)

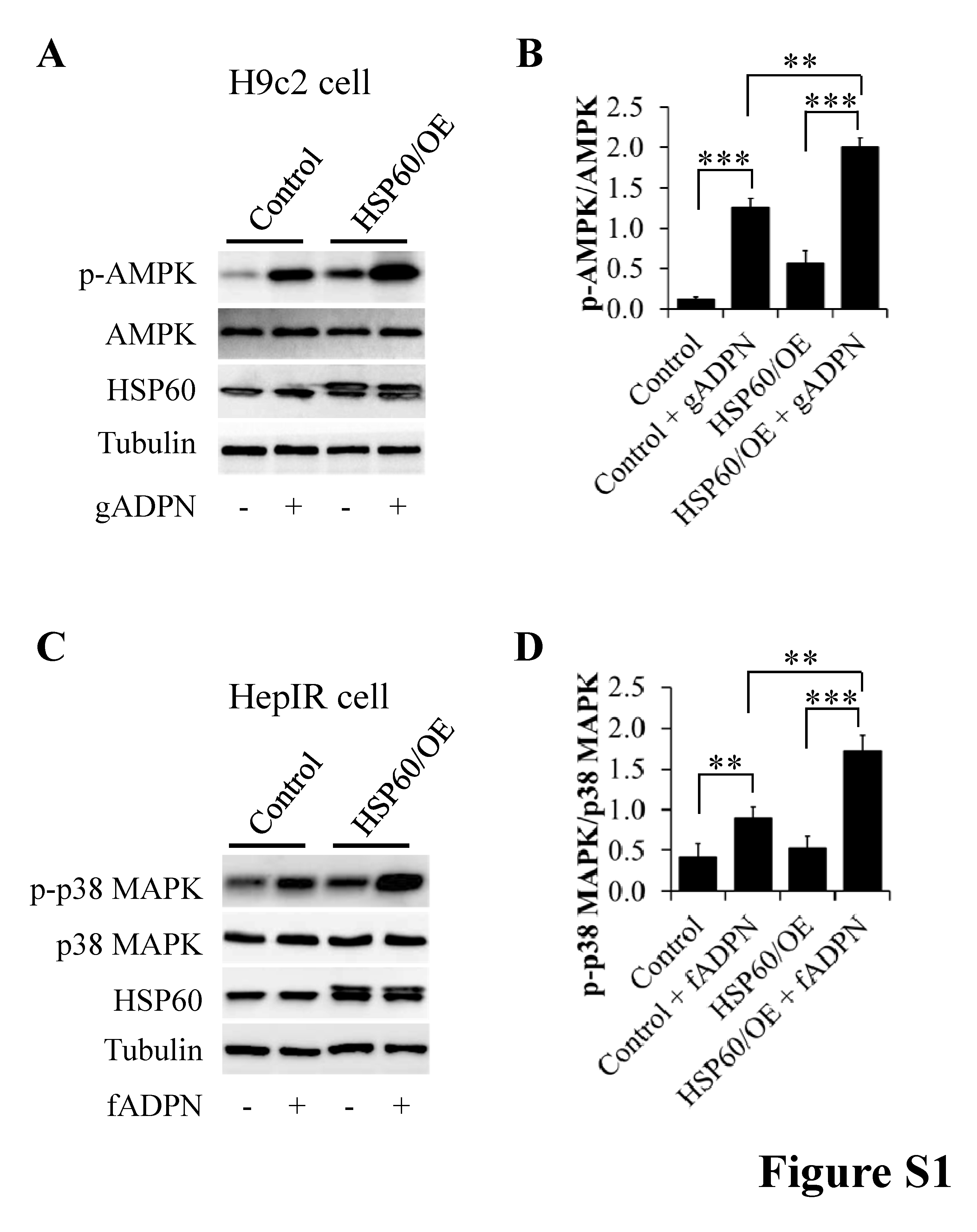

Supplement: Supplementary file 2 — Additional file 1 Figure S1. HSP60 overexpression enhanced adiponectin action. A Effects of HSP60 overexpression on adiponectin-stimulated phosphorylation of AMPK in H9c2 cells. B Quantification of phosphor-AMPK/AMPK in (A). C Effects of HSP60 overexpression on adiponectin-stimulated phosphorylation of p38 MAPK in HepIR cells. D Quantification of phosphor-p38 MAPK/p38 MAPK in (C). Results are mean ± SD. n = 4. **P < 0.01, ***P < 0.001 compared with the indicated group (one-way ANOVA). [file 12964_2020_546_MOESM2_ESM.tiff]

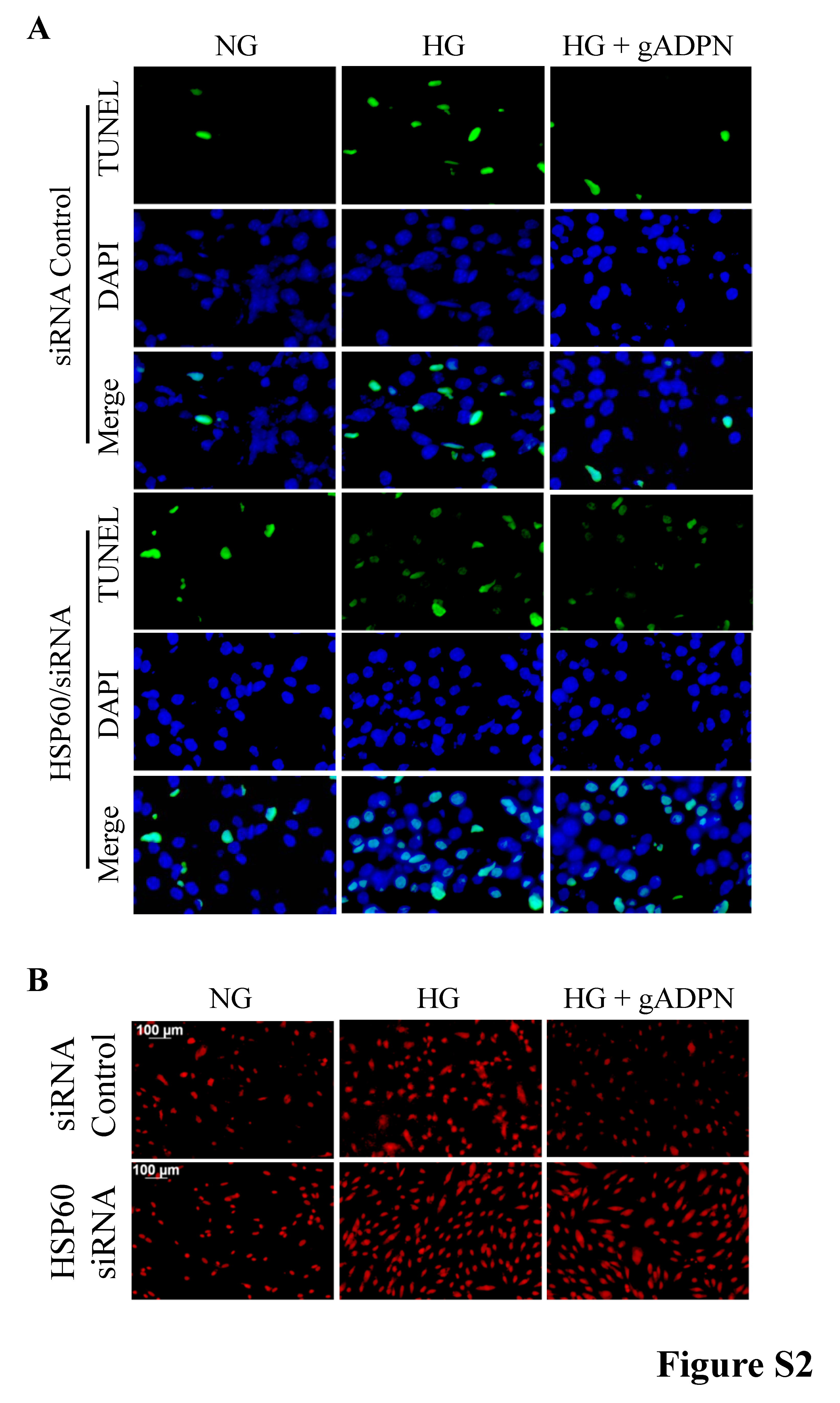

Supplement: Supplementary file 3 — Additional file 2: Figure S2. Effects of HSP60 knockdown on cell apoptosis and ROS formation in cardiac H9c2 cells. A Representative images showing the effects of HSP60 knockdown on apoptosis. B Representative images showing the effects of HSP60 knockdown on ROS formation. [file 12964_2020_546_MOESM3_ESM.tiff]

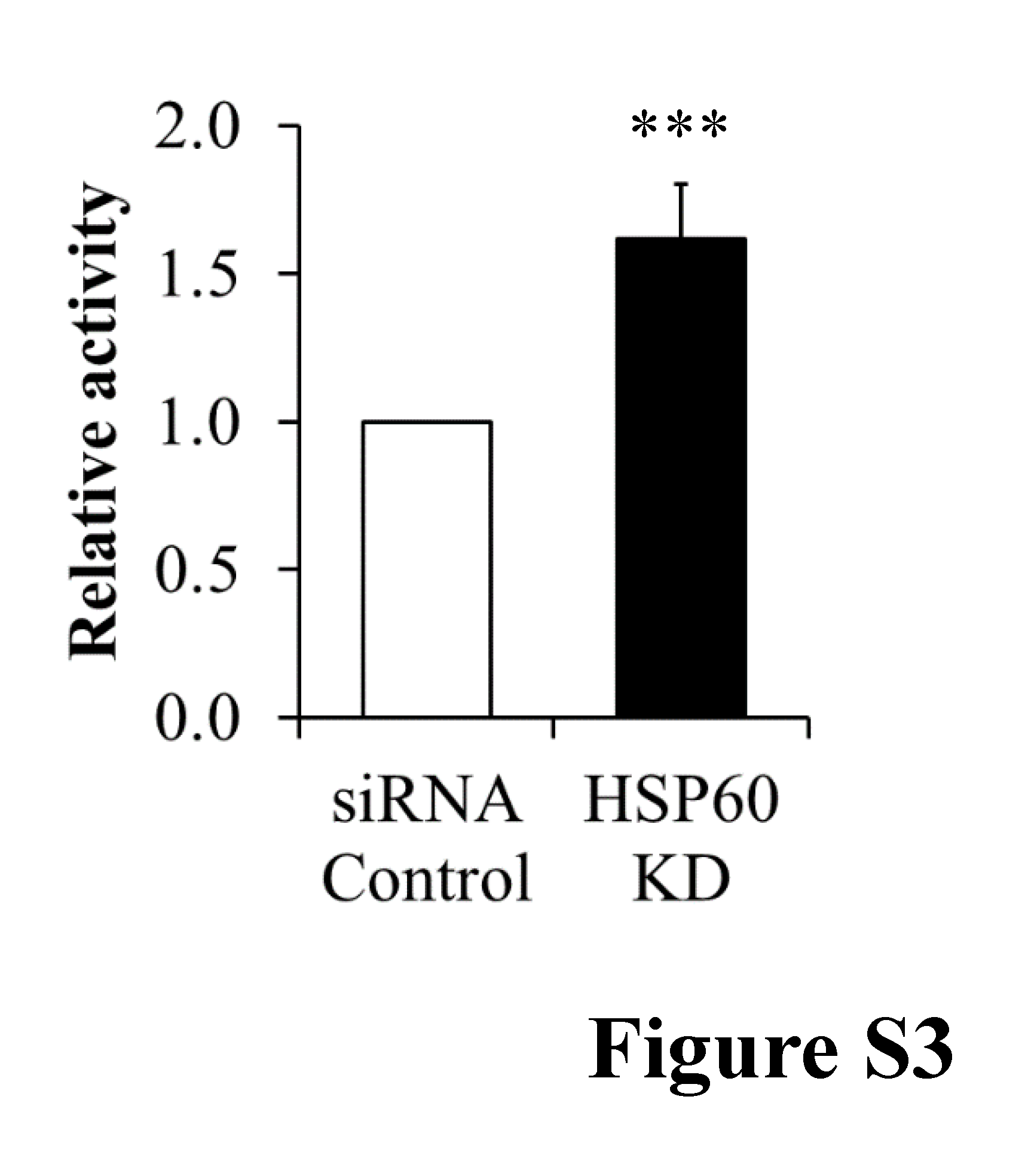

Supplement: Supplementary file 4 — Additional file 3: Figure S3. Effects of HSP60 knockdown on 20S proteasome activity in cardiac H9c2 cells. siRNA control and HSP60 KD cells were starved serum for 6 h. The chymotrypsin-like activity of 20S proteasome was determined using synthetic fluorogenic peptide substrate Suc-LLVY-AMC as described previously [52]. Results are mean ± SD. n = 4. ***P < 0.001 compared with the siRNA control group (one-way ANOVA). [file 12964_2020_546_MOESM4_ESM.tiff]
